# An Assay Suitable for High Throughput Screening of Anti-Influenza Drugs

**DOI:** 10.1371/journal.pone.0054070

**Published:** 2013-01-10

**Authors:** Lili Mao, Jun Wang, William F. DeGrado, Masayori Inouye

**Affiliations:** 1 Department of Biochemistry and Center for Advanced Biotechnology and Medicine, Robert Wood Johnson Medical School, Piscataway, New Jersey, United States of America; 2 Department of Pharmaceutical Chemistry, University of California San Francisco, San Francisco, California, United States of America; 3 Cardiovascular Research Institute, University of California San Francisco, San Francisco, California, United States of America; Centers for Disease Control and Prevention, United States of America

## Abstract

We developed a novel drug screening system for anti-influenza A virus by targeting the M2 proton channel. In the SPP (Single Protein Production) system, *E. coli* cell growth occurs only in the presence of effective M2 channel inhibitors, and thus simple measurement of cell growth was used as readouts for drug screening. Two potential inhibitors for M2 (V27A) mutant were verified using this method, which inhibit both the mutant and wild-type M2 channels.

## Introduction

Influenza A virus, which causes flu, is known as one of the most prevalent pathogenic viruses. Seasonal flu results in more than 50,000 deaths worldwide every year [1]. Pandemic flu caused over 50 million deaths in 1918 and about 22 million people infected in 2009 [2]. At present, the basic principles for defense of Influenza A virus are development of vaccines and antiviral drugs [3]. However, timely development of effective vaccines against new variants for each year is highly difficult and challenging, as influenza A virus is readily able to develop new variants resistant to new vaccines by simple genomic reassortment of hemaglutanin (HA) and neuroaminidase (NA). As a result, development of vaccines always lags behind the rate that a new subtype of Influenza A virus emerges. Although antiviral drugs is an alternative for effective treatment of influenza A virus infection, influenza A virus again readily develops drug resistance to these drugs by acquiring new mutations.

The most commonly used targets for antiviral drugs are M2 ion channel or neuraminidase encoded by the viral genome [4], [5]. M2 protein, which is crucial for viral infection, forms a tetrameric proton channel in the host cell membrane. Amantadine and rimantadine are well-known inhibitors to effectively block the wild type M2 proton channel and were used widely in the past for flu treatment [5]. But due to emergence of numerous M2 mutants, influenza A virus now is almost 100% resistant to these drugs. Here, we developed a novel high throughput screening system for agents to effectively block the M2 proton channel, thus to prevent viral infection.

## Results and Discussion

**Figure 1:**
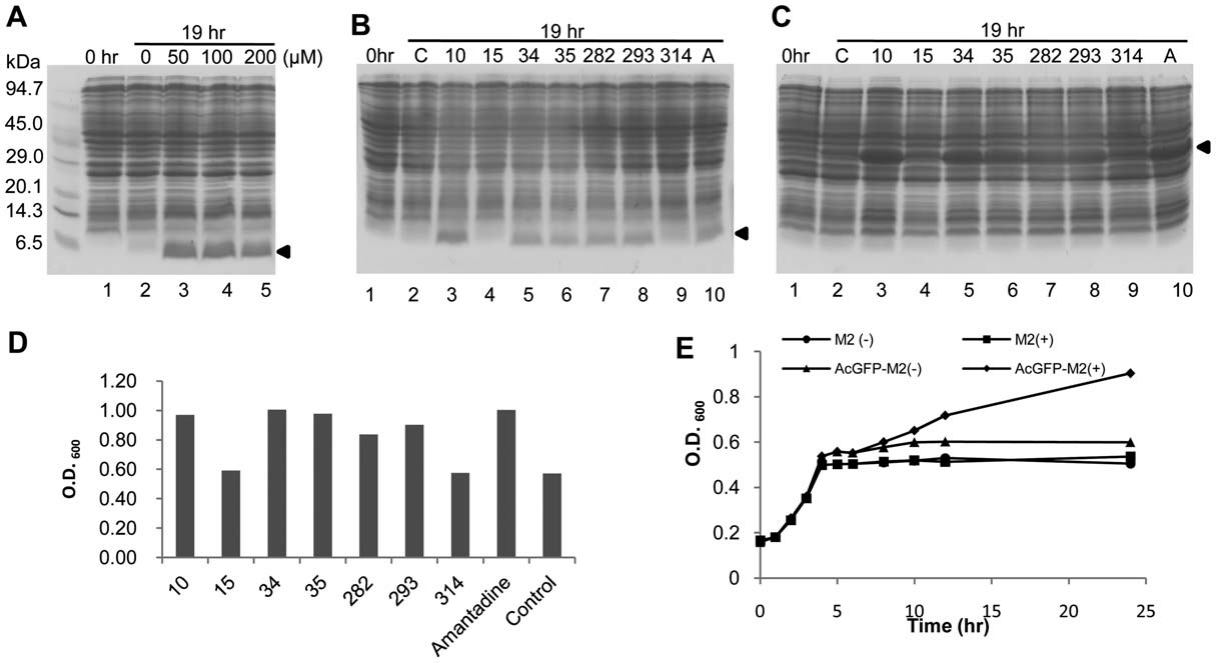
Expression of M2 protein in the SPP system. (**A**) E. coli cells harboring pColdII(sp-4) *m2* (from residue 2 to 49 of M2 protein) and pACYC*mazF* were grown at 37°C to OD_600_ = 0.5∼0.6, followed by cold-shock at 15°C for about 60 min. 1 mM of IPTG was added at 0 hr (Lane 1) for induction of M2 protein and MazF. Expression of M2 protein in the SPP system was examined in the presence of amantadine at different concentrations. Lane 2, 0 μM; Lane 3, 50 μM; Lane 4, 100 μM; Lane 5, 200 μM. After overnight incubation for 19 hours, cells from each culture were collected and subjected to SDS-PAGE. (**B**) Expression of M2 protein in the presence of other compounds besides amantadine. The final concentration of each compound in the culture is 50 μM. The experiments were carried out as described in (A). Lane 1, 1 mM IPTG is added to the culture at 0 hr, Lane 2: C, control without any additional compounds. Lane 3, compound 10, Lane 4, compound 15, Lane 5, compound 34, Lane 6, compound 35, Lane 7, compound 282, Lane 8, compound 293, Lane 9, compound 314, Lane 10, A, amantadine. (**C**) Expression of AcGFP-M2 fusion protein in the SPP system was carried out as described in (B). Positions of M2 protein and AcGFP-M2 fusion protein are indicated by arrowheads. (**D**) Cell density was measured as OD_600_ of each overnight culture that expressing AcGFP-M2 fusion protein, and plotted as histogram corresponding to the compounds added. (**E**) Growth curve of cultures to express M2 or AcGFP-M2 fusion protein. Cultures were started at 0 hr and the following experiment procedures are similar to that described in (A). OD _600_ of each culture is measured at every time point. M2 protein was induced at 5 hr with (▪) or without (•) amantadine. AcGFP-M2 fusion protein was induced at 5 hr with (♦) or without amantadine (▴).

The drug screening system was developed using the single protein production (SPP) system in *E. coli* [6], [7]. In the SPP system, the synthesis of all cellular proteins is inhibited by the induction of MazF, an ACA-specific mRNA interferase from *Escherichia coli*, except for the synthesis of a designated protein, of which mRNA is designed to have no ACA sequences [7]. Thus, an ACA-less gene coding for M2 protein (from residue 2 to 49 for M2 channel) was synthesized and cloned into pColdII(sp-4) [7]. Expression of M2 protein was toxic, and hence hardly detected (Figure 1A, lane 2), presumably because it disrupted the proton gradient across the membrane [8]. As a result, cells are unable to produce ATP and protein production is temporarily stopped. However, it was sufficiently well expressed to be easily detectable by SDS-PAGE in the presence of amantadine (Figure 1A, lanes 3, 4, and 5). This result clearly indicates that M2 protein is capable of forming an active proton channel, which is sensitive to amantadine. To validate this conclusion, we further tested seven other drugs for their inhibitory effect on M2 proton channel. Five of them were found to function as effective M2 channel inhibitors (Figure 1B). Similar results were observed with an N-terminal AcGFP-M2 fusion protein (Figure 1C). AcGFP is a green fluorescent protein from Aequorea coerulescens, which contains one residue substitution in its chromophore to make it much brighter than the native green fluorescent protein [9]. From these results, compounds 10, 34, 35, 282 and 293 were identified to be potential inhibitors of influenza A virus infection.

**Figure 2:**
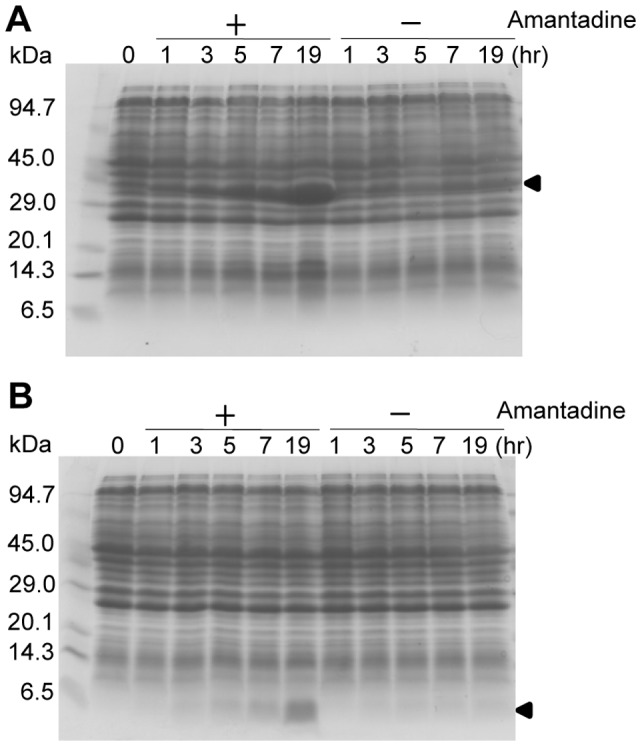
The expression profile of M2 protein and AcGFP-M2 fusion protein. (**A**) Expression of AcGFP-M2 fusion protein in the presence or absence of amantadine. Experiment procedures are the same as described in a. Positions of M2 protein and AcGFP-M2 fusion protein are indicated by arrowheads. (B) Expression of M2 protein in the presence or absence of amantadine. Cells harboring pColdII(sp-4) *m2* were cultured as described previously until O.D._600_ reached 0.5–0.6 and incubated at 15°C for 45 to 60 mins. 1 mM IPTG was added to at 5-hr and the culture was divided into two, one of which contains 50 μM amantadine. Cells from 1.5 ml culture were collected at 0 hr, 1 hr, 3 hr, 5 hr, 7 hr, and 19 hr after induction and subjected to SDS-PAGE assay.

In the SPP system, cell growth is completely inhibited, while cells are metabolically active as they are at the quasi-dormancy state so that side effects of drugs on cell growth can be excluded. Surprisingly, although AcGFP fails to be as a reporter since it is not fluorescent in the AcGFP-M2 fusion protein, we found in the present manuscript that cell growth is resumed as measured by the increase of cell density using O.D._600_, when AcGFP-M2 fusion protein is expressed in the presence of inhibitors for the M2 channel activity (Figure 1D). Significant increases of the cell density were observed in the cultures, in which compounds 10, 34, 35, 282, 293 and amantadine were added, while compounds 15 and 314 were unable to resume cell growth as the control experiment without addition of any drugs. In a time-course experiment (Figure 1E), M2 protein or AcGFP-M2 fusion protein was induced together with MazF at 5 hrs by the addition of 1 mM IPTG in the presence or absence of 50 μM amantadine. To our surprise, cell growth was resumed 3 hrs after induction only in the culture containing amantadine to produce AcGFP-M2 (shown by arrows). The amount of AcGFP-M2 further increased for the next 16 hrs in the culture with amantadine, while no significant changes in protein patterns were observed in the other culture without the addition of amantadine (Figure 2A), Expression of M2 protein was observed only in the presence of amantadine and accumulated gradually during the 19-hr induction time (Figure 2B).

**Table 1:** Inhibition of M2 channel activity in Oocyte assay.

Compounds	WT (%)	V27A (%)
10	95	0
15	0	n.a
34	89	53.4
35	92	29.7
180	90.4	82.7
189	95.9	25.2
206	89.1	94.5
282	95.7	3.4
293	93.2	12.4
314	69.2	10.9
332	10.6	6.3
343	3.8	6.6
352	20.3	7.2
360	12.9	17.4
362	14.1	40
Amantadine	90.8	0

The inhibition efficiency of each compound was examined at the concentration of 100 μM. The values in the table represent how much the M2 channel activity is inhibited. WT, wild type M2 channel. V27A, M2 channel with V27 A mutation. The Oocyte assay was conducted as described previously [12].

**Figure 3:**
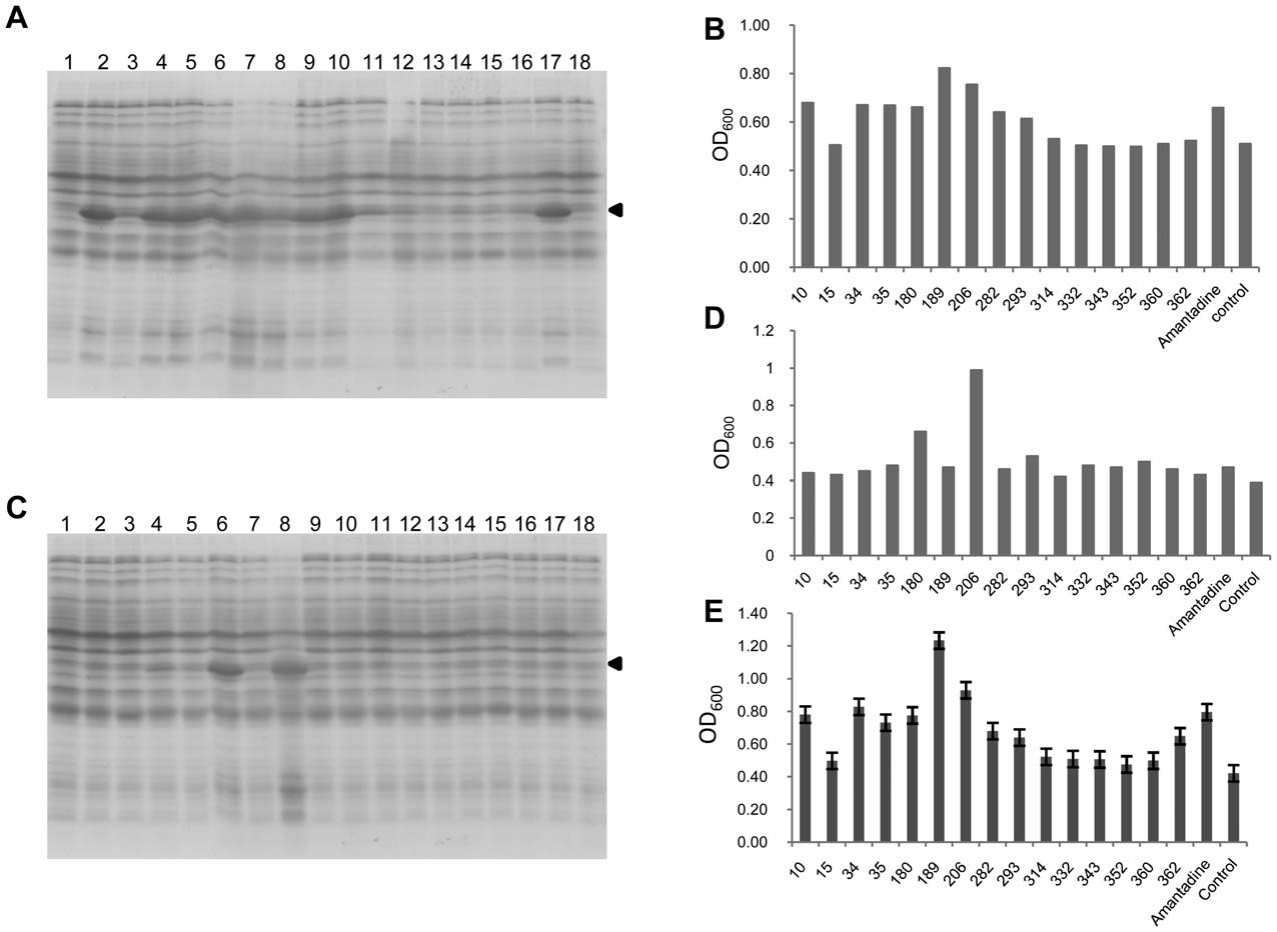
Screening for the inhibitors of M2 proton channel. (**A**) Expression of AcGFP-M2 protein in the presence of a group of compounds to be screened. Experiment procedure is the same as described in Figure 1A. Lane 1, 1 mM IPTG is added to the culture at 0 hr, Lane 2, compound 10, Lane 3, compound 15, Lane 4, compound 34, Lane 5, compound 35, Lane 6, compound 180, Lane 7, compound 189, Lane 8, compound 206, Lane 9, compound 282, Lane 10, compound 293, Lane 11, compound 314, Lane 12, compound 332, Lane 13, compound 343, Lane 14, compound 352, Lane 15, compound 360, Lane 16, compound 360, Lane 17, amantadine, Lane 18, control without any additional compounds. (**C**) Expression of AcGFP-M2 (V27A) fusion protein in the presence of a group of compounds to be screened as described in (A). Positions of AcGFP-M2 fusion protein are indicated by arrowheads. (**B**) and (**D**), Cell density was measured as OD_600_ of each overnight culture that expressing AcGFP-M2 fusion protein, and plotted as histogram corresponding to the compounds added. (**E**) After adding 1 mM IPTG for induction of AcGFP-M2 fusion protein, 100 μl of cell culture was loaded to a 96-well plate, in which a certain compound is added to each well (n = 3). OD_600_ was collected after overnight incubation. All the compounds' concentration was 50 μM.

We further tested the inhibitory effect of other eight compounds on the production of AcGFP-M2 and found that out of these compounds, eight compounds (10, 34, 35, 180, 189, 206, 282 and 293) were able to resume the synthesis of the fusion protein (Figure 3A). Consistently with the result with amantadine, cell growth was resumed after overnight incubation with all these eight compounds as in the case of amantadine (Figure 3B).

Next, we tested if the same compounds are able to inhibit the M2 (V27A) mutant channel, which is known to be resistant to amantadine [10], [11]. The V27A mutation was introduced to pColdIIAcGFP-M2 (sp-4) by site-directed PCR mutagenesis. It was found that Ac-GFP-M2 (V27A) was well expressed only in the presence of compound 180 or 206 (Figure 3C). Subsequent O.D._600_ measurements confirmed that only the SPP expression systems that contain compounds 180 and 206 displayed significant cell growth resumption (Figure 3D).

Similar results were obtained by electrophysiology assay in *Xenopus laevis* oocyte. As summarized in tabe1, Compound 180 and 206 appear to be the best two inhibitors for M2 (V27A) mutant ion channel, which exhibit 82.7% and 94.5% inhibition of channel conductivity, respectively (Tabel 1). The eight compounds (10, 34, 35, 180, 189, 206, 282 and 293), which was identified above as potential inhibitors of M2 channel, show around 90% or more inhibition of the wild type M2 ion channel conductivity in the electrophysiology assay. A few compounds were also tested in plaque reduction assay as reported earlier [12], [13]. Compound 34 and 35 significantly inhibit wild type A/M2 virus replication at 5 µM, comparable to the potency of amantadine at the same concentration. In consistent with the electrophysiology assay, compound 34 also inhibits the replication of virus harboring A/M2-V27A mutation when tested at 50 µM [12]. Another two compounds 180 and 206, which are more potent than 34 and 35 in inhibiting V27A in electrophysiology assay, show higher viral inhibition activities at 10 µM. Remarkably, the most potent compound 206 completely inhibits V27A virus replication at 5 µM concentration [13]. Compound 34 and 35 correspond to compound 8 and 9, respectively, in Balannik V. *et* *al*., 2009. Compound 180 and 206 correspond to compound 1 and 9, respectively, in *Wang J. et* *al*., 2011.

Subsequently, screening was carried out by using 96-well plates. A 100-ml culture of the SPP system harboring pColdII AcGFP-M2 (sp-4) was grown to O.D._600_ = 0.5∼0.6. After cold-shock at 15°C for 60 min, 1 mM of IPTG was added to the culture for induction of AcGFP-M2 fusion protein. A hundred μl of the culture was distributed to each well of 96-well plates and individual compounds were added to the wells. After overnight incubation at 15°C with vigorous shaking, O.D._600_ was measured using micro plate reader (Molecular devices spectramax 384 plus). A similar set of compounds as previously isolated were observed to be potential inhibitors for M2 proton channel since cell growth in these wells were detected (Figure 3E).

It was previously reported that expression of M2 in bacteria would result in retarded cell growth because the host cell membrane became permeable by the M2 channel [14]. However, our results demonstrate for the first time a high correlation between the rate of growth resumption and the expression of AcGFP-M2 protein in the SPP system. The reason that causes growth resumption in the SPP system is still unknown. It has been confirmed that cells after growth resumption still contain the plasmid which produces MazF and MazF retains its toxicity (data not shown). It is suspected that some fragments from AcGFP-M2 fusion protein may play a role in neutralizing the toxicity of MazF. Nevertheless, this screening method using *E. coli* provides an extremely simple, easy and fast assay system to screen anti-influenza drugs in comparison with tedious patch clamping assay systems to measure the channel conductivity [15]. Using this method we identified a number of channel inhibitors, which can be potentially used as anti-influenza drugs. This high throughput screening system may be extended for screening of drugs against ion channel proteins from other RNA and DNA viruses such as Vpu protein form HIV-1 and p7 from HCV [16], [17].

## Materials and Methods

### Strains, plasmids and medium

The ACA-less gene for M2 (from residue 2–49) was synthesized with flanked NdeI and BamHI restrictions sites in pUC57 by Genscript. pColdII *m2* was constructed by transferring M2 gene to pColdII(sp-4) with the former two restriction sites. pColdII*acGFP-m2* was constructed by first inserting acGFP into pColdII (sp-4) by restriction enzymes SacI and BamHI, followed by inserting m2 gene by restriction enzymes HindIII and XbaI. The linker sequence between AcGFP and M2 protein is “GSEFKL” which comes from restriction sites remained on pCold vector. Mutations of M2 genes were obtained by site-directed PCR mutagenesis. All the assays were carried out on *E. coli* strain BL21 (DE3) and cultured in M9 minimal medium.

### Compounds

Amantadine was purchased from Sigma-Aldrich laboratories. All the other tested drugs were provided by Dr. William F. DeGrado.

### Cell growth

Cells with pColdIIm2 or pColdacGFP-m2 were incubated on 37°C and grown until O.D._600_ reached 0.5–0.6. The culture was then chilled on ice for five minutes and incubated on a 15°C shaker for 45 to 60 mins, after which 1 mM IPTG was added. Thereafter, the culture was divided to test tubes and 96-well plates that contain different treatments to be screened. The culture volume is 5 ml for test tubes and 100 μl for 96-well plates. Induction of M2 protein is continued for overnight (∼19 hr) on 15°C. Cells from 1.5 ml culture were collected and subjected to SDS-PAGE assay. O.D._600_ readings of 96-well plates were recorded by Molecular devices spectramax 384 plus.
